# Reduction of Carbon Dioxide in Filtering Facepiece Respirators with an Active-Venting System: A Computational Study

**DOI:** 10.1371/journal.pone.0130306

**Published:** 2015-06-26

**Authors:** Erik Birgersson, Ee Ho Tang, Wei Liang Jerome Lee, Kwok Jiang Sak

**Affiliations:** 1 Department of Chemical and Biomolecular Engineering, National University of Singapore, Singapore; 2 ST Dynamics, 249 Jalan Boon Lay, Singapore; Northwestern Polytechnical University, CHINA

## Abstract

During expiration, the carbon dioxide (CO_2_) levels inside the dead space of a filtering facepiece respirator (FFR) increase significantly above the ambient concentration. To reduce the CO_2_ concentration inside the dead space, we attach an active lightweight venting system (AVS) comprising a one-way valve, a blower and a battery in a housing to a FFR. The achieved reduction is quantified with a computational-fluid-dynamics model that considers conservation of mass, momentum and the dilute species, CO_2_, inside the FFR with and without the AVS. The results suggest that the AVS can reduce the CO_2_ levels inside the dead space at the end of expiration to around 0.4% as compared to a standard FFR, for which the CO_2_ levels during expiration reach the same concentration as that of the expired alveolar air at around 5%. In particular, during inspiration, the average CO_2_ volume fraction drops to near-to ambient levels of around 0.08% with the AVS. Overall, the time-averaged CO_2_ volume fractions inside the dead space for the standard FFR and the one with AVS are around 3% and 0.3% respectively. Further, the ability of the AVS to vent the dead-space air in the form of a jet into the ambient – similar to the jets arising from natural expiration without a FFR – ensures that the expired air is removed and diluted more efficiently than a standard FFR.

## Introduction

The form and function of the human respiratory system ensures an efficient gas exchange with the environment. During expiration, hot, humid air that is rich in carbon dioxide (CO_2_) and depleted in oxygen is vented away from the body in the form of one jet from the mouth or two jets from the nostrils that entrains air from the surrounding; during inspiration, air from the immediate surroundings of the face is inhaled in a diffuse manner from all directions in front of the mouth/nostrils. This combination of directed expiration and diffuse inspiration ensures that rebreathing is kept to a minimum, because we inhale air close to our faces and exhale far away and in a manner that dilutes the expired air. Respiration thus also significantly changes the airflow pattern around us. Now, when we put on a facepiece respirator (FFR) covering our mouth and nostrils, we alter the air exchange by not only extending the physiological dead space of the respiratory system by the dead-space volume of the FFR itself, but also by altering the airflow pattern during respiration.

In this context, it is therefore perhaps not surprising that FFRs are known to adversely affect the comfort of the wearer in terms of, for example, lowered thermal comfort [1–3] and elevated CO_2_ levels [4,5]. The decreased thermal comfort is mainly due to increased humidity and temperature inside the dead space of the FFR from respiration and thermal regulation, which together give rise to a significantly higher apparent temperature than the ambient under normal conditions; apparent temperatures around 53°C have been measured by Roberge et al. [3] for ambient conditions of 22°C and 24% relative humidity. The elevated CO_2_ levels originate from the CO_2_-rich expired air that is around one-hundred times higher in CO_2_ concentration than that of atmospheric air; CO_2_ volume fractions of around 3.0 ± 0.5% [6] have been measured for subjects carrying out low-intensity exercise inside the dead space of a FFR. Especially the significantly increased CO_2_ concentration is potentially hazardous: increased CO_2_ levels have been linked to changes in visual performance, altered exercise endurance, headaches, shortness of breath, decreased reasoning and alertness as well as increased irritability [7].

**Fig 1:**
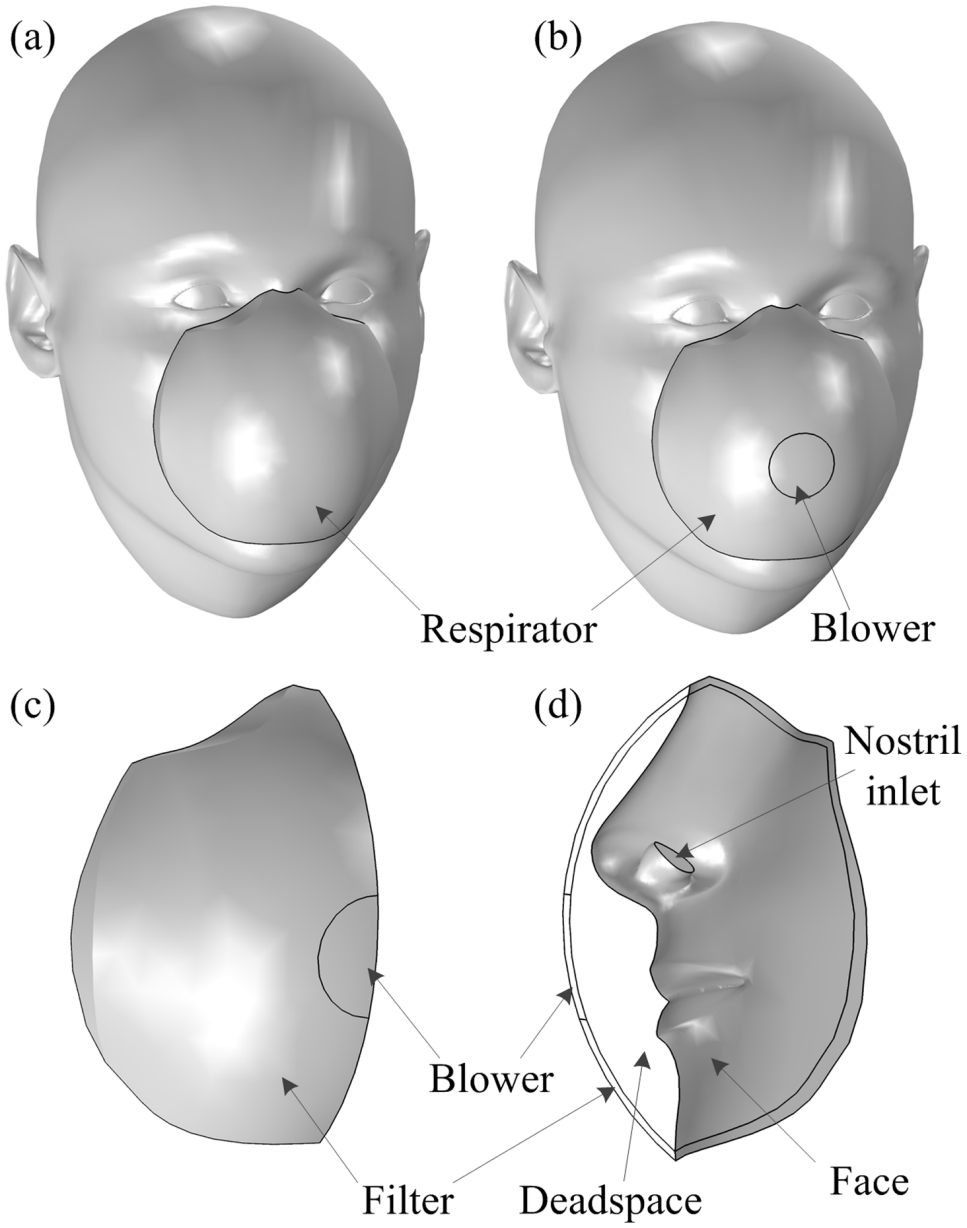
Illustration of FFRs and the computational domains. (a) a standard FFR, (b) a FFR equipped with the blower of the AVS and (c-d) the computational domains comprising the dead space of the FFR, the filter and the blower; for the simulation of the standard FFR, the blower region was treated as a filter.

In order to reduce the CO_2_ levels in the dead space to near-ambient levels, we introduce an active venting system (AVS) that aims to mimic and thus restore the functionality of the human respiratory system even when a FFR is covering our mouth and nostrils. In short, the lightweight AVS comprises a housing for a one-way valve, a blower and battery that can be attached to the FFR with negligible mechanical deformation of the filter. The blower illustrated in Fig 1 vents the air out from the FFR and should thus reduce the CO_2_ concentration inside the dead space.

We quantify the reduction by solving a computational-fluid-dynamics (CFD) model for conservation of mass, momentum and the dilute species CO_2_ together with constitutive relations for the respiratory cycle, filter and blower in the dead space between the face and the FFR as well as the filter and blower. The surrounding of the FFR and the likelihood of rebreathing CO_2_ is then discussed in terms of simple scaling arguments. Here, the key advantage of solving a standard CFD model for passive transport is the high spatial and temporal resolution that can be achieved, whereas corresponding experimental measurements are usually carried out for average quantities [3,7]. As a point of reference, we validate our simulations with existing experimental measurements for a standard FFR.

## Mathematical Formulation

We consider a three-dimensional representation of the dead space and filter of a FFR as well as the blower for the AVS-equipped FFR as depicted in Fig 1c and 1d based on a computer-aided design of the human head [8] and additions carried out in Solidworks. Here, we have assumed that the face and mask are symmetric, whence we only need to consider half of the domain in Fig 1a and 1b.

The physical phenomena associated with respiration and passive transport of CO_2_ are captured via the equations of change for turbulent and laminar momentum in the dead space and filter respectively and for mass and the species CO_2_, boundary conditions and constitutive relations for the breathing cycle, expired carbon dioxide, filter and blower. These are based on the following main underlying assumptions and characteristics:

1. CO_2_ is dilute since its volume fraction ranges from around 0.04% in the ambient to around 5% in the alveolar air.
2. The ambient is well ventilated, such that any CO_2_ vented out from the FFR is removed and not rebreathed—we shall later discuss this condition in detail.
3. The flow is isothermal, because the temperature variations, Δ*T* ∼ 15 K, arising from respiration are less than the total ambient temperature, *T*_amb_ ∼ 300 K; i.e. Δ*T* << *T*_*amb*_
4. The pressure variations in the dead space of the FFR and across the filter are small compared to the ambient pressure, because the pressure drop, Δ*p*, across the filter for the air flow is ∼ 30 Pa for the conditions considered here; i.e., Δ*p* << *p*_*amb*_ ∼ 10^5^ Pa.
5. The flow is incompressible and the air behaves as an ideal gas.
6. In view of the above, the volume fraction is equal to the molar fraction.
7. The flow pattern can be captured with the standard *k*-*ε* turbulent model in the dead space and with the Brinkman equation in the filter.
8. Quiet breathing through the nostrils for a young adult man as summarized by Guyton et al. [9].
9. The transition of expired CO_2_ from the anatomic dead space to the alveolar air can be described with linear functions.
10. The physiological dead space is equivalent to the anatomic dead space in the respiratory system.
11. The respiratory cycle follows that described by Lee et al. [10].
12. The blower of the AVS is represented by a boundary condition.
13. The one-way valve that is part of the AVS is not resolved explicitly to reduce the degree of complexity; instead, the impact of the one-way valve on the fan characteristic curve is assumed accounted for by increasing the driving voltage of the blower, thus compensating for the increased pressure drop in the system.

While several of these assumptions could easily be relaxed or removed, they allow for the derivation of a relatively simple mathematical model that captures all the leading-order phenomena associated with the salient features of breathing in a FFR with and without AVS.

### Governing equations

In the dead space of the FFR, we solve for incompressible, turbulent flow described by the standard *k*-*ε* model [15]:

∇⋅u=0,ρ∂u∂t+ρ(u⋅∇) u=∇⋅[−pI+(μ+μt)(∇u+(∇u)T)−23ρkI],ρ∂k∂t+ρ(u⋅∇) k=∇⋅[(μ+μtσk)∇k]+Pk−ρε,ρ∂ε∂t+ρ(u⋅∇) ε=∇⋅[(μ+μtσε)∇ε]+Cε1εkPk−Cε2ρε2k, (1)

Where

μt=ρCμk2ε,Pk=μt[∇u  :  (∇u+(∇u)T)]. (2)

Here, *ρ* is the density of the air (kg m^−3^), **u** is the air velocity (m s^−1^), *t* is the time (s), *p* is the pressure (Pa), *μ* and *μ*_*t*_ are the dynamic and turbulent viscosities (Pa s), *k* is the turbulent kinetic energy (m^2^ s^−2^), *ε* is the turbulent dissipation (m^2^ s^−3^) and σ_*k*_, σ_*ε*_, *C*_*μ*_, *C*_ε1_ and *C*_ε2_ are adjustable parameters (1).

In the filter, we solve for laminar flow in a porous medium, given by [16]

∇⋅u=0,ργ∂u∂t=∇⋅[−pI+μγ(∇u+(∇u)T)]−μκu, (3)

where *κ* is the permeability (m^2^) and *γ* is the porosity (1). In the plain fluid, described by the *k*-*ε* model, the dependent variables and parameters are Reynold's averaged quantities, whereas the corresponding counterparts in the porous medium are volume-averaged quantities.

Throughout the dead space and the filter, we solve for transport of dilute CO_2_:

$$\frac{\partial \varphi }{\partial t}+\nabla \cdot \left(u\varphi \right)=D^{eff}\nabla^{2}\varphi ,$$ (4)

with *ϕ* denoting the volume fraction of CO_2_ (1); the effective diffusivity coefficient, *D* ^*eff*^, is defined as

$$D^{eff}=\{\begin{array}{cc} D_{CO_{2}}+D_{t} & \text{in the dead space and blower,}\\ \gamma^{\frac{3}{2}}D_{CO_{2}} & \text{in the filter.} \end{array}$$ (5)

The turbulent diffusivity, *D*_*t*_, is described by

$$D_{t}=\frac{\mu_{t}}{\rho \text{S}{\text{c}}_{t}},$$ (6)

where Sc_t_ is the turbulent Schmidt number (1). The effective diffusion coefficient in the filter has been described with a standard Bruggeman relation with $D_{CO_{2}}$ denoting the molecular diffusion (m^2^s^−1^) of CO_2_ in air.

### Boundary and initial conditions

At the nostril inlet, we prescribe the airflow velocity, turbulent intensity and length scale:

$$u\cdot n=-{\dot{V}}_{\text{resp}}/A_{\text{nose}};\;I_{t}=I_{\text{nose}};\;l_{t}=l_{\text{nose}};$$ (7)

and either fully developed flow during inspiration

∇φ⋅n=0, (8)

or the volume fraction during expiration of CO_2_ as

$$\varphi =\varphi_{\text{exp}}.$$ (9)

Here, **n** is the unit normal pointing out of the domain, ${\dot{V}}_{resp}$ is the air flow from the respiration (m^3^ s^−1^), *I*_*t*_ and *l*_*t*_ are the turbulent intensity and length scales respectively, *A*_nose_ is the cross-sectional area of the nostrils, and *ϕ*_*exp*_ is the volume fraction of CO_2_ (1) from the respiration.

At the walls, we prescribe standard wall functions and zero flux of CO_2_, and at the exterior of the filter, we specify the gauge pressure

p=0, (10)

and either fully developed flow during expiration

∇φ⋅n=0, (11)

or the volume fraction during inspiration of CO_2_ as

$$\varphi =\varphi_{\text{amb}},$$ (12)

where *ϕ*_*amb*_ is the volume fraction of CO_2_ of the ambient.

For the FFR equipped with the AVS, we prescribe the following boundary condition for the blower:

$$p=-p_{\text{fan}}.$$ (13)

The minus sign on the RHS of Eq 13 results in a negative gauge pressure inside the FFR, which corresponds to the fan sucking the air out.

The initial conditions for *t* = 0 are set as follows:

$$u=0;\;p=0;\;\varphi =\varphi_{0}.$$ (14)

The initial volume fraction of CO_2_, *ϕ*_0_, was found from the steady periodic behavior of the solution, as we shall see later. The initial values of the turbulent kinetic energy and dissipation were computed by the software COMSOL 4.4 (see *Numerics* for more details).

### Respiration

**Fig 2:**
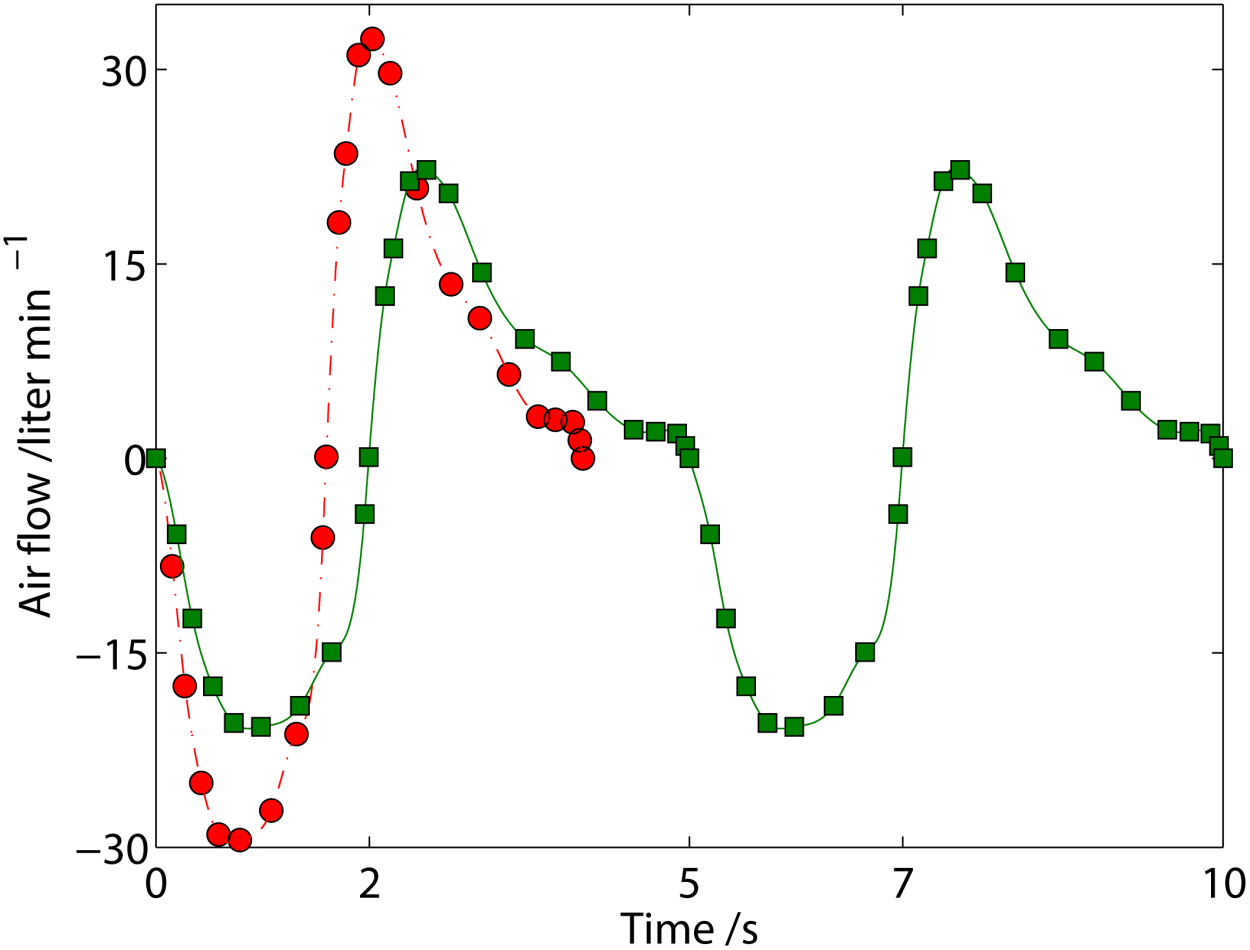
Airflow for quiet breathing of a young man. (●) experiments^(6)^ for a tidal volume of 0.58 liter at a breathing frequency of 15 breaths min^−1^ and (−) cubic spline interpolation thereof; (■) breathing pattern adjusted for a tidal volume of 0.5 liter at a breathing frequency of 12 breaths min^−1^and (−) cubic spline interpolation thereof. (*N*.*B*.: The breathing pattern can easily be extended beyond the ten seconds shown here.)

In order to capture the air flow, ${\dot{V}}_{resp}$, from the nostrils as a function of time, we start with the experiments for quiet breathing by Lee et al. [10], who measured the inspiration (1.6 s duration) and expiration (2.4 s duration) for a breathing frequency of 15 breaths min^−1^ and a tidal volume of 0.58 liter as shown in Fig 2.

For our respiratory cycle with a breathing frequency of 12 breaths min^−1^ and a tidal volume of 0.5 liter, we therefore multiply the time series for the measured air flow with the factor 15/12 to obtain the total period for respiration, *t*_resp_, of 5 s with 2 s and 3 s for inspiration and expiration respectively. We further multiply the magnitude of the air flow during inspiration and expiration with one constant each to reduce the tidal volume to 0.5 liter, which results in the breathing pattern depicted for 10 s in Fig 2. Finally, the volume flow of air at any time is determined via interpolation with cubic splines between the data points.

### Filter

For the filter, we determine the permeability numerically such that the filter resistance is 10 mmAq for airflow of 85 liter min^−1^and a surface area of 100 cm^2^.

### Blower

The fan characteristic curve for the centrifugal blower of the AVS is fitted to measurements by the manufacturer Risun Corp. [11], which results in the following functional form for the static fan pressure, *p*_fan_ (Pa), and the volume flow through the blower, ${\dot{V}}_{fan}$ (m^3^s^−1^):

$$p_{\text{fan}}=(-1.6216e5\times {\dot{V}}_{\text{fan}}+67.1)\left[(-1.6216e5\times {\dot{V}}_{\text{fan}}+67.1)>0\right].$$ (15)

(Note that we have not included the units of the constants for the sake of brevity.) The volume flow through the fan is determined by integrating the air flow over the blower:

$${\dot{V}}_{\text{fan}}=2\underset{A_{\text{fan}}/2}{\int \int }u\cdot ndA.$$ (16)

The factor 2 is included since we only consider half of the geometry and blower; see Fig 1c and 1d.

### Carbon dioxide

**Fig 3:**
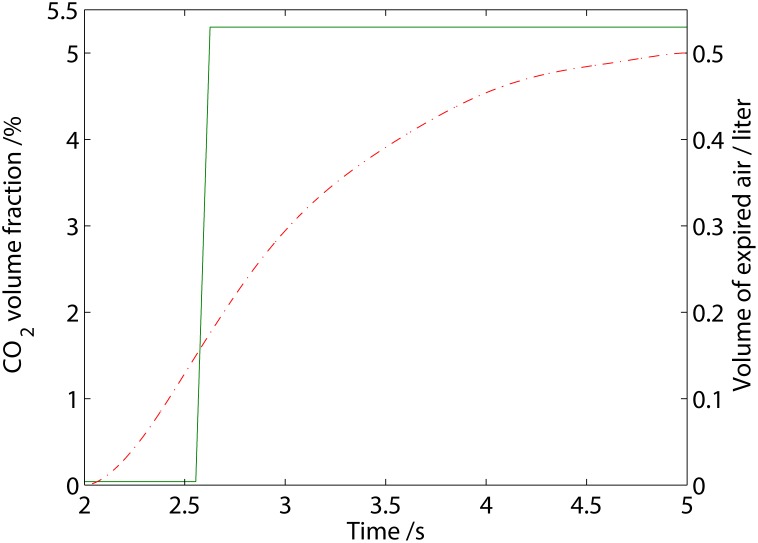
The derived function for (−) the CO_2_ volume fraction during expiration as well as (−) the total volume of expired air as a function of time.

In the ambient, we assume a volume fraction of 0.04% of CO_2_ and well-ventilated conditions such that any expired CO_2_ is removed once it is vented out of the FFR. For the CO_2_ originating from expiration, we derive a function that is illustrated in Fig 3.

In essence, air from the anatomical dead space is expired first with a CO_2_ volume fraction of the ambient, followed by a transition period from the ambient CO_2_ level to that of the alveolar air, and finally alveolar air rich with CO_2_, which can be written as

$$\begin{array}{c} \varphi_{\text{exp}}=\varphi_{\text{amb}}(M(t)<t_{\text{pd}})+((\varphi_{\text{alv}}-\varphi_{\text{amb}})/t_{\text{mix}}(M(t)-t_{\text{pd}})+\varphi_{\text{amb}})(M\geq t_{\text{pd}})\left[M(t)<(t_{\text{mix}}+t_{\text{pd}})\right]+\\ \varphi_{\text{alv}}(M(t)\geq (t_{\text{mix}}+t_{\text{pd}})), \end{array}$$ (17)

where *M(t)* is the modulus, defined as

$$M(t)=\text{Mod}(t,\;t_{\text{resp}}).$$ (18)

The parameters, *t*_*pd*_ and *t*_*mix*_, were fitted to ensure that

$$\int {}_{M(t_{ins})}^{M(t_{resp})}{\dot{V}}_{\text{exp}}\varphi_{\text{exp}}dt=V_{{\text{CO}}_{2}},$$ (19)

and

$$\int {\;}_{M(t_{ins})}^{M(t_{pd})}{\dot{V}}_{\text{exp}}dt=V_{\text{ads}};$$ (20)

here, $V_{CO_{2}}$ is the volume of CO_2_ from one expiration, *V*_ads_ is the volume of the anatomic dead space of the respiratory system and ${\dot{V}}_{\text{exp}}$ is the airflow during expiration, i.e. the air flow with a positive magnitude in Fig 2. The end-tidal CO_2_ in our derived expression is similar to that measured by Zhao et al. [12].

## Numerics

The CFD model for passive transport of CO_2_ inside a FFR was implemented in the commercial finite-element software Comsol Multiphysics 4.4. Around 10^6^ elements, amounting to ∼10^6^ degrees of freedom for the geometries with and without AVS were employed to ensure mesh-independent solutions for the dependent variables **u**, *p*, *k*, *ε* and *ϕ*. The parameters are summarized in Table 1. In particular, we note that the volume of the dead space, *V*_ds_, for the FFR is 0.1 liter and that the cross-sectional area of the nostrils is 0.98 cm^2^, which is close to the measured average of around 1 cm^2^ by Gupta et al. [13]. Further, we note that the fitted permeability is of the same order of magnitude as that employed by Lei et al. [14].

**Table 1:** Summary of parameters.

Parameterb	Value and unit	Source
**Air**		
C*_μ_*	0.09	[17]
C*_ε1_*	1.44	[17]
C*_ε2_*	1.92	[17]
σ*_k_*	1.0	[17]
σ*_ε_*	1.3	[17]
*p_amb_*	1 atm	estimated
*ρ*	1.1 kg m^−3^	estimated
*μ*	1.9×10^−5^ Pa s	[17]
*ϕ_amb_*	0.04%	estimated
*ϕ*0 (with AVS)	0.43%	calculated
*ϕ*0 (without AVS)	5.15%	calculated
Sct	0.71	estimated
$D_{CO_{2}}$	1.7×10^−5^ m^2^s^−1^	[17]
**Filter**		
*κ*	3.43×10^−11^ m^2^	fitted
γ	0.88	[14]
*A*filter (without AVS)	137.5 cm^2^	measured
*A*filter (with AVS)	132 cm^2^	measured
*V*ds	0.1 liter	measured
**Blower**		
*A*fan	5.5 cm^2^	measured
**Respiration**		
*V*tid	0.5 liter	[9]
*ν*	12 breaths min^−1^	[9]
*V*ads	0.15 liter	[9]
*ϕ_alv_*	5.3%	[9]
$V_{CO_{2}}$	0.018 liter	[9]
*t*pd	2.555 s	fitted
*t*mix	7.122×10^−2^ s	fitted
*t*resp	5 s	[9]
*t*ins	2 s	[9]
*t*exp	3 s	[9]
*I*nose	1%	[14]
*I*nose	1 cm	estimated
*A*nose	0.98 cm^2^	measured

## Results and Discussion

**Fig 4:**
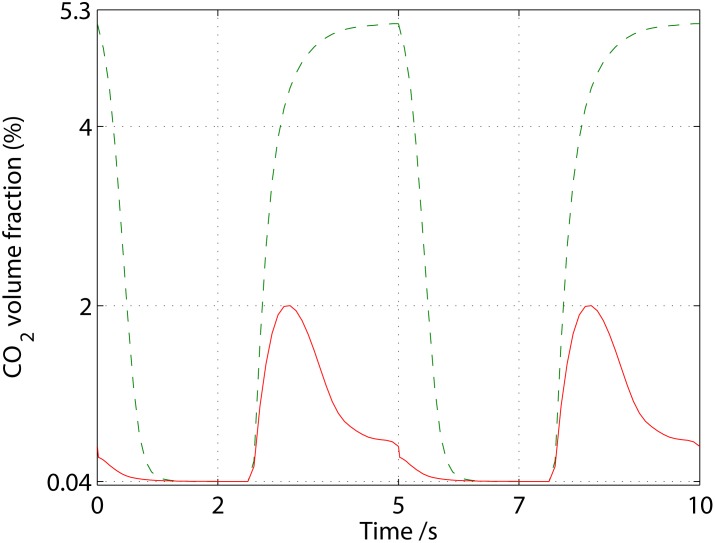
Volume-averaged volume fraction (%) of CO_2_ in the dead space of a FPR without (- -) and with (−) an AVS for well-ventilated ambient conditions.

As alluded to in the *Introduction*, we expect the CO_2_ concentration for respiration in a standard FFR to increase significantly above that of the ambient—this is indeed the case as can be inferred from Fig 4.

In particular, the volume-averaged CO_2_ volume fraction, defined as

$$\bar{\varphi }(t)\equiv \frac{2}{V_{\text{ds}}}{\int \int \int }_{V_{\text{ds}}/2}\varphi (t)dV,$$ (21)

has risen to around 5.2% at the end of expiration in the dead space of the standard FFR; let's call this volume fraction ${\bar{\varphi }}_{\text{end}}$. During inspiration, the entire dead-space volume, *V*_ds_, with its high CO_2_ concentration is inspired after which the dead space fills up with ambient air around half-way through the inspiration. In other words, one can quickly estimate the volume fraction of CO_2_ that is inspired from

$$\varphi_{\text{ins}}=\frac{{\bar{\varphi }}_{\text{end}}V_{\text{ds}}+\varphi_{\text{amb}}(V_{\text{tid}}-V_{\text{ds}})}{V_{\text{tid}}}\approx \frac{{\bar{\varphi }}_{\text{end}}V_{\text{ds}}}{V_{\text{tid}}}\approx 1\text{ \%.}$$ (22)

Here, we have removed the contribution from the ambient, because it is roughly one-hundred times lower than the CO_2_ volume fraction in the dead space at the end of expiration. In contrast and as a reference, inspiration without a FFR would lead to *ϕ*_ins_ = *ϕ*_amb_ = 0.04%; i.e. the mask adds 1% to the inspired CO_2_ content. From these estimates, it is also clear that the internal volume of a standard FFR should be as small as possible to reduce inspiration of trapped CO_2_ in its dead space.

If we proceed by taking the time average of the volume-averaged volume fraction, defined as

$$\frac{1}{t_{\text{resp}}}\int {}_{0}^{t_{\text{resp}}}\bar{\varphi }(t)dt,$$ (23)

we find a time- and volume-averaged CO_2_ volume fraction of 3%, which is within the bounds of experimental measurements by Roberge et al. [6] and Sinkule et al. [7] for low-intensity physical activity with a FFR.

**Fig 5:**
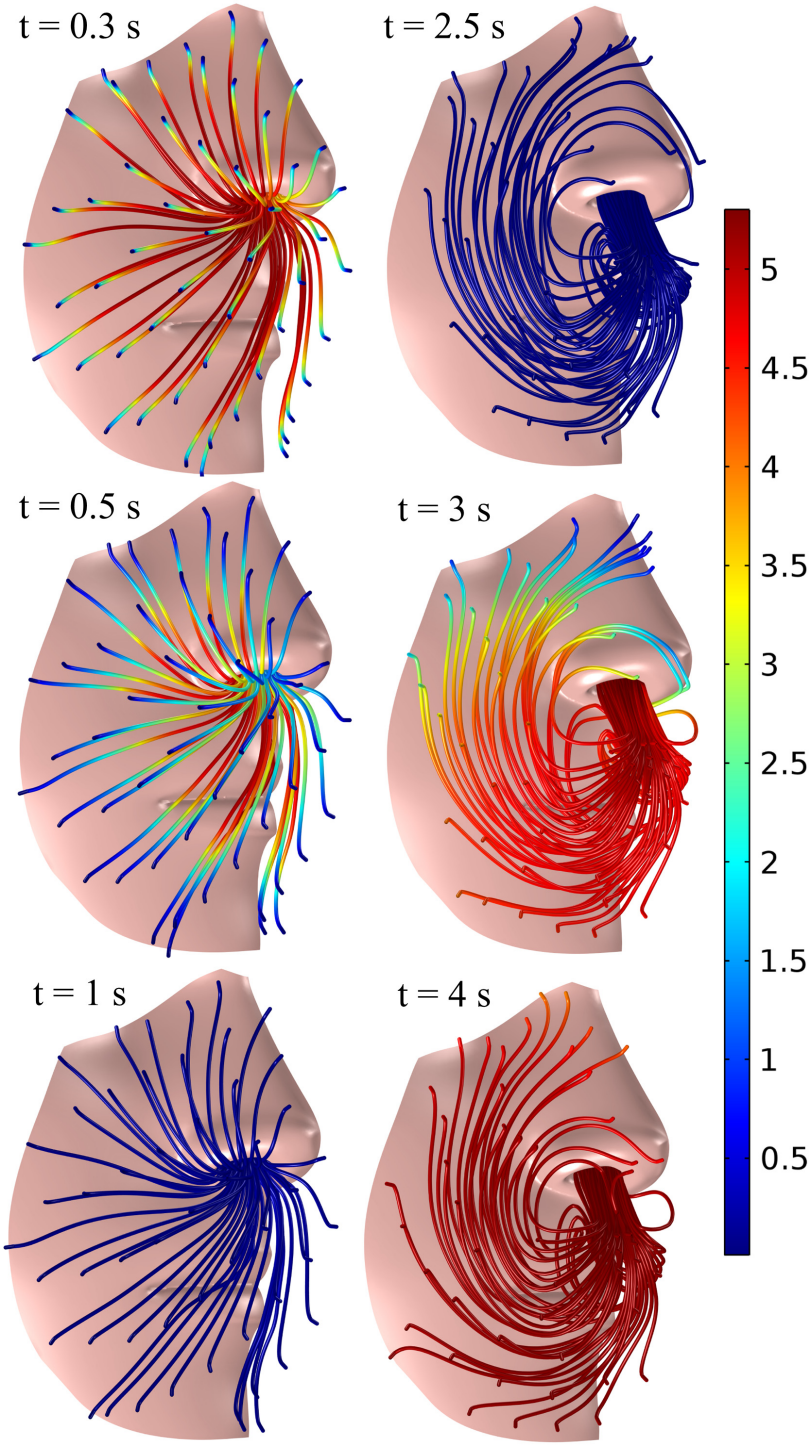
Streamlines of the airflow during inspiration at 0.3, 0.5 and 1 s and during expiration at 2.5, 3 and 4 s for a standard FFR. The volume fraction of CO_2_ is shown in color with a range of 0.04% (blue) to 5.3% (red).

Turning our attention to the local flow pattern and CO_2_ levels inside the mask in Fig 5, we find that during inspiration that lasts for 2 seconds, air from the ambient is sucked in more or less uniformly from all around the mask. At 1 s, i.e. half-way through the inspiration, the alveolar air has been almost completely replaced with ambient air. The flow pattern then changes significantly for the subsequent expiration that lasts 3 seconds: the expired jet from the nostril impinges on the filter and is then vented out all around the filter, which results in a steadily increasing CO_2_ concentration. The dead space is filled with alveolar CO_2_ at around 4 seconds. Clearly, the reason for the high levels of CO_2_ in a standard mask is because the jet—which is supposed to vent the air away from us—is stopped and the expired air vented in a diffusive manner.

**Fig 6:**
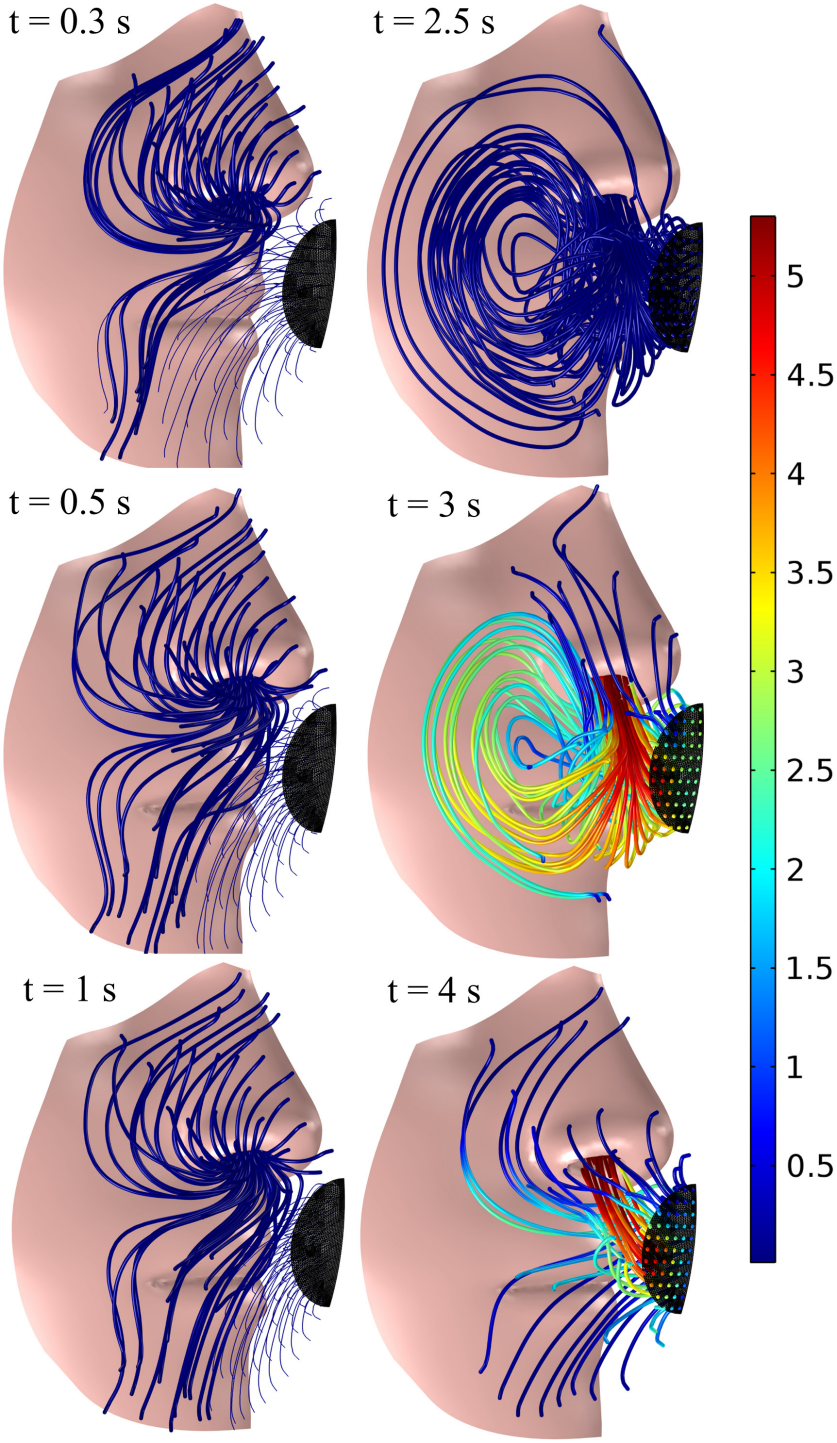
Streamlines of the airflow during inspiration at 0.3, 0.5 and 1 s and during expiration at 2.5, 3 and 4 s for the FFR equipped with the AVS. The volume fraction of CO_2_ is shown in color with a range of 0.04% (blue) to 5.3% (red).

Once we add the AVS with a view to restore the functionality of respiration, we reduce the CO_2_ level significantly as expected in Fig 4: the CO_2_ levels do no increase monotonically during expiration, as was the case for the standard FFR, but rather peak around 2% and then drop towards 0.4% at the end of expiration. The AVS has thus managed to reduce the amount of CO_2_ that remains in the dead space by around ten times; the time- and volume-averaged CO_2_ fraction has dropped from around 3% for the standard mask to 0.3% for the mask with the AVS. This reduction is significantly higher than the measured reduction of CO_2_ by around 1% for an exhalation valve by Sinkule et al. [7] (albeit measured at a slightly higher frequency of breathing at 12.9 breaths min^-1^ and a tidal volume of 0.92 liter). Somewhat unexpected though, is that the CO_2_ concentration inside the mask increases to around 2% during expiration even with the AVS installed. In order to find the answer, let us observe the local flow pattern and CO_2_ concentration inside the dead space in Fig 6 in more detail.

During expiration, we see that the jet circulates at around 2.5 to 3 seconds inside the mask, before being sucked out by the blower. There is thus an initial increase in CO_2_ concentration for the peak airflow occurring at around 3 seconds, which corresponds to the expiratory peak in Fig 2, after which the expired jet is sucked out directly by the blower around 4 seconds. The main reason for the observed circulation is the placement of the blower, which does not allow the entire impinging jet to be vented immediately—this could be remedied by introducing an air guide for the expired jet. Now, having said that, the blower still manages to reduce the CO_2_ concentration at the end of expiration inside the dead space by around ten times, resulting in an inspired CO_2_ that is correspondingly ten times less than that of a standard FFR. Returning to Eq 22, we find the corresponding inspired CO_2_ volume fraction to be 0.08% with the AVS, which is close to ambient levels.

**Fig 7:**
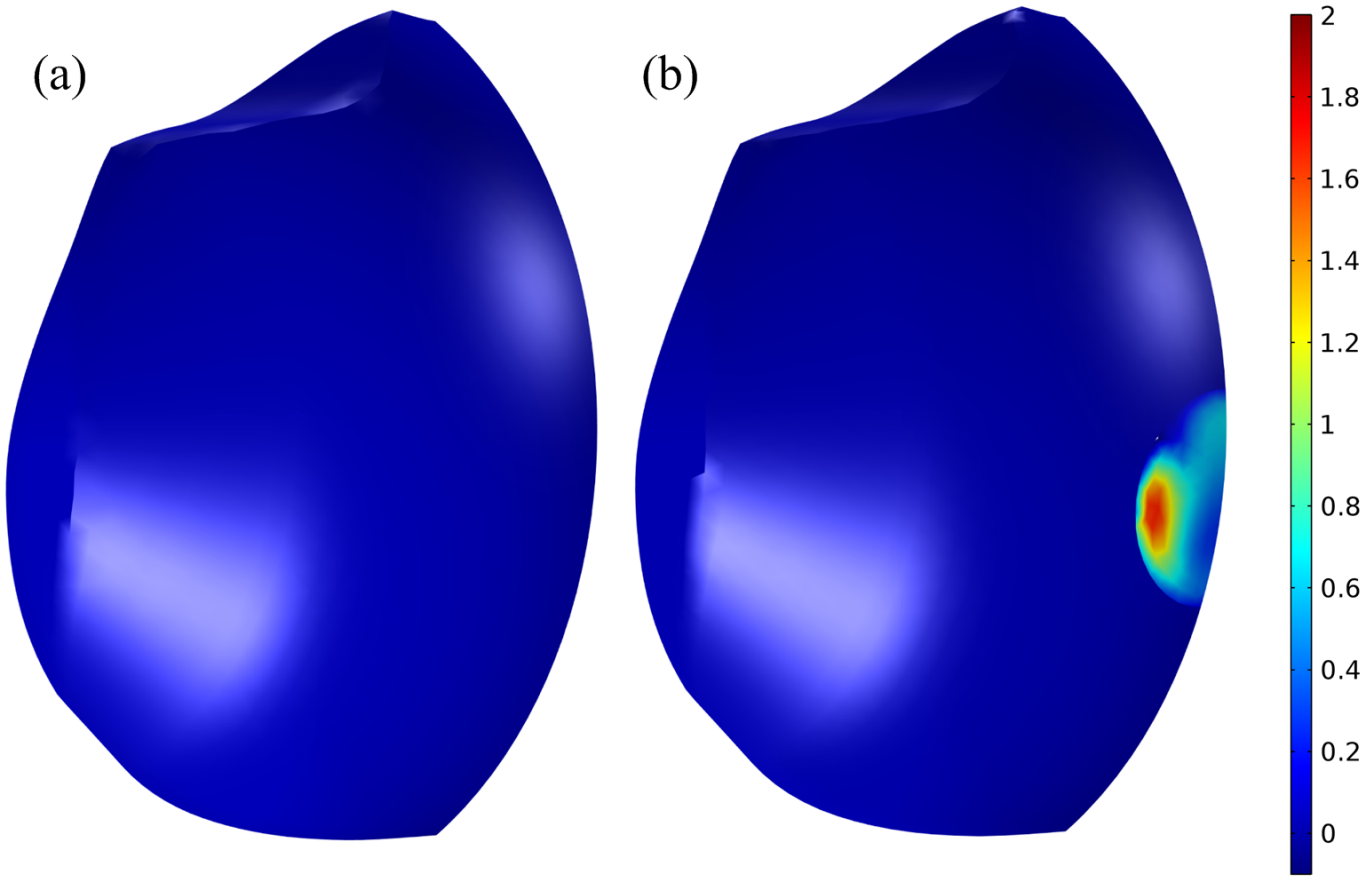
The normal velocity (m s^−1^) during expiration at 3 seconds for (a) a standard FFR and (b) a FFR equipped with the AVS. The average speed out of the filter into the ambient of the standard FFR is ∼10^−2^ m s^−1^; the speed of the expired air through the blower for the AVS is ∼1 m s^−1^.

We have so far assumed that the expired air is completely removed and replaced with fresh, ambient air during inspiration in our mathematical model. For conditions where this assumption is no longer valid, such as stagnant air, we need to carefully reassess our findings. For this purpose, let us again consider the physiology of breathing: During expiration, a human vents the air in the form of jets either from the nostrils or from the mouth with a speed of ∼ 1 m s^−1^. This ensures that the expired air—with a relative humidity close to 100%, an average CO_2_ level of around 3–4% depending on the physical activity, and a temperature of around 36°C—is transported sufficiently far away that the next inspiration takes mainly fresh air from the ambient; during inspiration, the air flows from the entire surrounding into either the nostrils or the mouth at a lower local velocity than the jets during expiration. When a standard FFR is worn, those jets are damped out inside the dead space as we saw earlier and expired more or less evenly through the filter with a velocity that is around 100-times smaller (depending on the filter resistance and area) than that of the aforementioned jets as depicted in Fig 7a, in which $\left|u\cdot n\right|∼10^{-2}$ m s^−1^ for the airflow through the filter. At these speeds, the expired air will not travel far away from the mask's exterior.

It is therefore reasonable to assume that during the subsequent inspiration, some of the expired air is likely to be inhaled again. We can estimate the speed of the expired air out from the filter *a priori* to calculations by considering conservation of total mass:

$$\rho {\dot{V}}_{\text{resp}}∼\rho \left|u\cdot n\right|\;A_{\text{filter}},$$ (24)

in which the left-hand side represent the mass flowing in from the nostrils and the right-hand side represents the mass flowing out through the filter of a standard FFR. Rearranging yields

$$\left|u\cdot n\right|∼\frac{{\dot{V}}_{\text{resp}}}{A_{\text{filter}}}∼\frac{20{\text{ liter min}}^{-1}}{100{\text{ cm}}^{2}}∼10^{-2}{\text{ m s}}^{-1}.$$ (25)

Given the low airflow speed through the FFR, the expired air is therefore likely to be found in a hemisphere-shaped volume centered on the FFR for stagnant air conditions. In contrast, the blower of the AVS replicates the natural mechanism and physiology of breathing in the sense that it pulls the expired air inside the dead space and vents it in the form of a jet with |u⋅n|∼1 m s^−1^into the surrounding, as can be seen from the significantly higher air speed in the blower in Fig 7b. The AVS should thus significantly reduce the likelihood of rebreathing compared to a standard FFR.

## Conclusions

We have carried out CFD simulations for quiet breathing through a standard FFR and one equipped with an AVS. In short, the model comprises the equations of change for momentum, mass and CO_2_ for turbulent flow inside the dead space of a FFR and blower and laminar flow inside the filter together with the necessary constitutive relations as well as initial and boundary conditions. The model allows for the visualization of the airflow during respiration coupled with quantification of the CO_2_ levels in space and time with a high resolution, as opposed to time- and volume-averages in experiments.

In agreement with experimental observations, the standard FFR gives rise to a time- and volume-averaged CO_2_ volume fraction of around 3%. For the FFR with the AVS, the CFD simulations suggest that the AVS is able to lower the time- and volume-averaged CO_2_ volume fraction to 0.3%, which is a near-to ten-fold reduction; and that the AVS reduces the average inspired CO_2_ to 0.08%, which is close to the ambient level. While this reduction has yet to be validated with experiments, the base case with a standard FFR for passive transport of CO_2_ agrees well with experiments; this, together with the fact that the treatment of blowers and passive species transport is well established and standard in CFD, lends credibility to the findings.

In addition, and as expected, the blower of the AVS—which seeks to mimic nature by sucking the air out and away from the FFR—should reduce the likelihood of rebreathing CO_2_ as compared to the standard FFR.

Finally, we note that the model can be modified to account for various breathing patterns, ambient conditions and different types of FFRs and blowers.
